# Supplementary material for: SORLA regulates endosomal trafficking and oncogenic fitness of HER2
Source: Nat Commun. 2019 May 28;10:2340. doi: 10.1038/s41467-019-10275-0 (PMC6538630; doi:10.1038/s41467-019-10275-0)
Supplement: Supplementary file 1 — Supplementary Information [file 41467_2019_10275_MOESM1_ESM.pdf]

SORLA regulates endosomal trafficking and oncogenic fitness of HER2

Supplementary information:

- Supplementary Figures 1-8 and associated legends
- Supplementary Table 1. Antibodies used in this study
- Supplementary Table 2. Oligonucleotide sequences of siRNA and shRNA used in this study

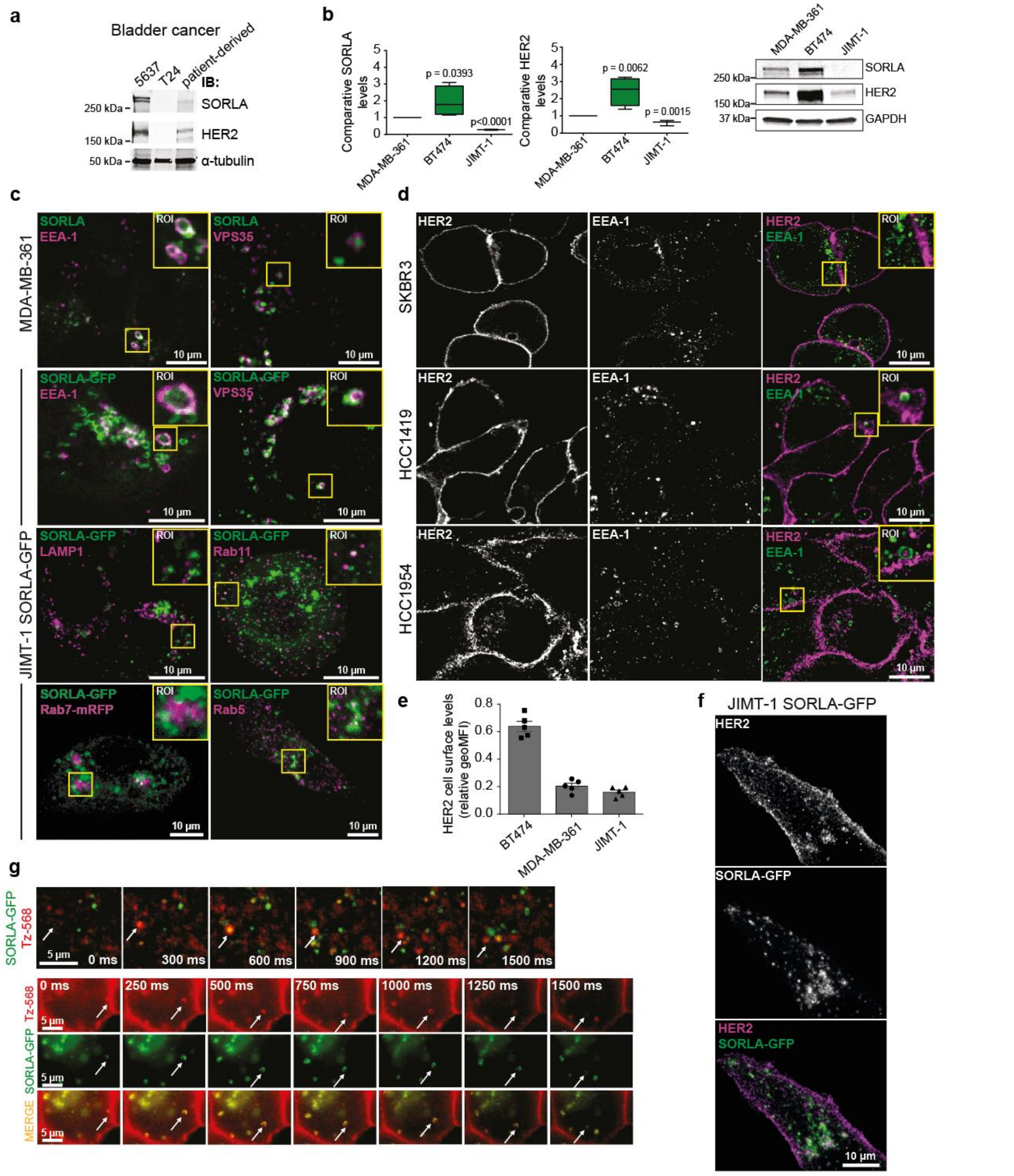

Supplementary Figure 1. (**a**) Western blot analysis of SORLA and HER2 levels in bladder cancer cell lines (5637 and T24) and a patient-derived cell culture. α-tubulin is a loading control. (**b**) Representative western blots and quantification of total SORLA and HER2 protein levels in the indicated cells lines. GAPDH was blotted to control for loading. MDA-MB-361 (n=4), BT474 (n=4) and JIMT-1 (n=3). Values are normalized to MDA-MB-361 levels (data are mean ± s.d., unpaired Student's *t*-test). (**c**) Confocal microscopy imaging of endogenous SORLA or SORLA-GFP (green) with endogenous EEA1, VPS35,

LAMP1, Rab11, Rab5 (magenta) or Rab7-mRFP (magenta; Rab7 was overexpressed due to lack of reliable Rab7 antibodies for staining) in MDA-MB-361 (top panel) or JIMT-1 cells. (**d**) Endogenous HER2 (magenta) and EEA1 (green) staining in SKBR3, HCC1419 and HCC1954 cells. (**e**) Flow cytometry analysis of HER2 cell-surface levels in BT474, MDA-MB-361, and JIMT-1 cells (n = 5 from 4 independent experiments). (**f**) Co-localization between SORLA-GFP and HER2 in JIMT-1 cells (Pearson's R = 0.3±0.01, n = 130 cells from 7 independent experiments). Details of colocalization analysis can be found in the methods section. (**g**) Live-cell TIRF imaging (top panel) and confocal imaging (bottom panel) of AlexaFluor-568-labelled trastuzumab (Tz-568; red) and SORLA-GFP (green) in MDA-MB-361 cells.

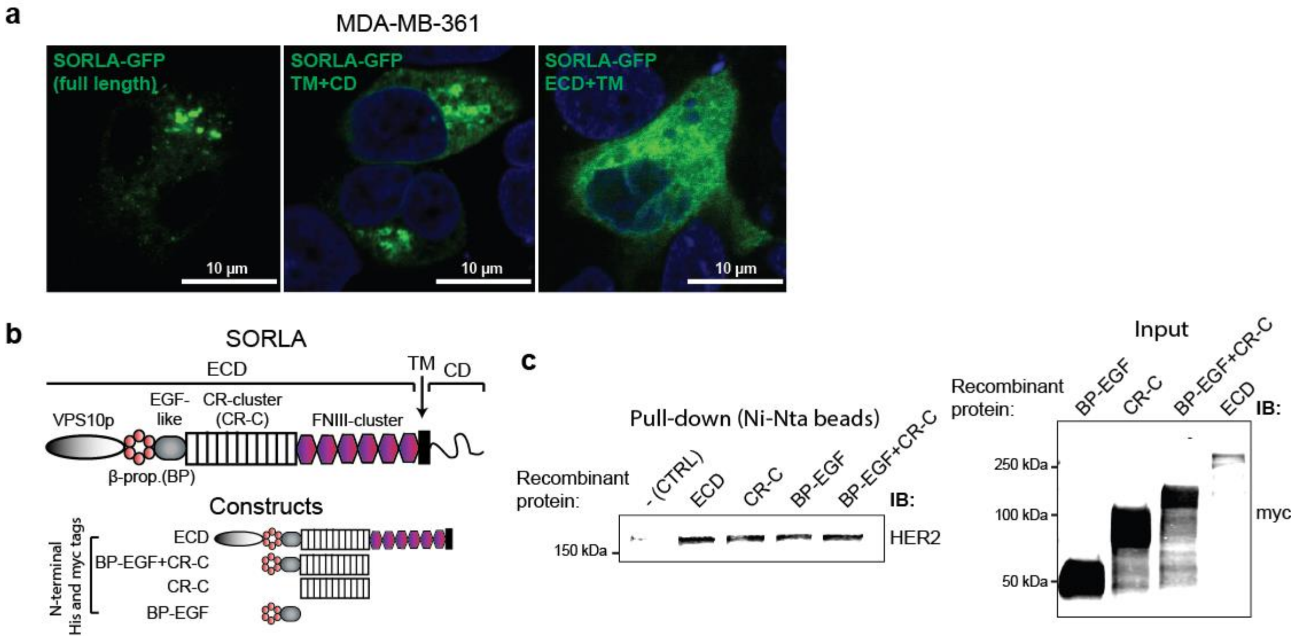

Supplementary Figure 2. (**a**) Confocal imaging of different SORLA-GFP fusion proteins in MDA-MB-361 cells. ECD: extracellular domain; TM: transmembrane domain; CD: cytosolic domain. (**b**) Schematic of the SORLA protein domains and summary of the constructs used. (**c**) Pull-down of endogenous HER2 from BT474 cell lysate with His-tagged ECD fragments of SORLA (from b) captured on Ni-Nta beads (left panel). Input of purified ECD fragments recognized with a myc antibody are also shown (right panel).

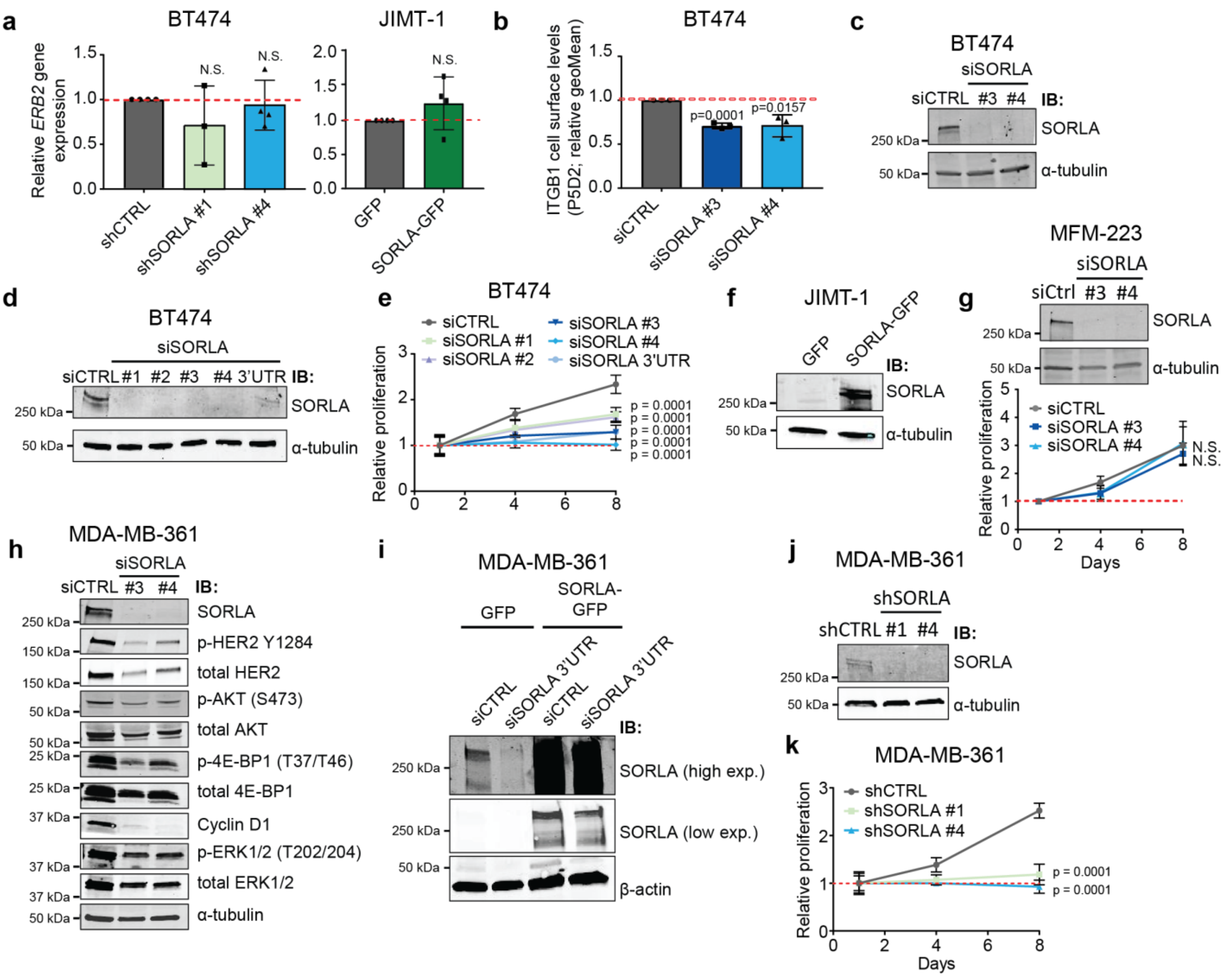

Supplementary Figure 3. (**a**) Quantitative real-time PCR analysis of *ErbB2* mRNA gene expression level in the indicated transfectants. Data are mean ± s.d. (n = 4 independent experiments; statistical analysis: unpaired Student's *t*-test). (**b**) Flow cytometry analysis of ITGB1 levels on the cell surface (detected with P5D2 antibody) in BT474 cells after silencing with control (siCTRL) and SORLA (siSORLA #3 and #4) siRNAs (mean ± s.d. of n = 3 independent experiments; statistical analysis: unpaired Student's *t*-test). (**c-d**) Western blot analysis of SORLA in scramble (siCTRL) and SORLA (siSORLA #1, siSORLA #2, siSORLA #3, siSORLA #4 and siRNA targeting the SORLA 3´UTR, siSORLA 3´UTR) silenced BT474 cells. α-tubulin is a loading control. (**e**) Proliferation of BT474 cells after SORLA silencing as in (d). Data are mean ± s.d (n = 4 independent experiments; statistical analysis: two-way ANOVA). (**f**) Western blot analysis of SORLA in JIMT-1-GFP and JIMT-1-SORLA-GFP cells. α-tubulin is a loading control. (**g**) Proliferation of scramble (siCTRL) and siSORLA (siSORLA#3, siSORLA#4) silenced non-HER2-amplified MFM-223 breast cancer cells. Proliferation data are mean ± s.d. (n = 12 (4 replicates, 3 independent experiments); statistical analysis: two-way ANOVA). (**h**) Western blot analysis of the indicated signalling proteins (phosphorylated and total) in MDA-MB-361 cells after scramble (siCTRL)

and SORLA silencing (siSORLA #3 and siSORLA #4), α-tubulin is a loading control. Shown are representative immunoblots from n = 2 independent experiments. (**i**) Western blot analysis of SORLA in scramble (siCTRL) and SORLA (siSORLA 3´UTR) silenced MDA-MB-361-GFP and MDA-MB-361-SORLA-GFP cells. Shown are two exposures for SORLA. β-actin is a loading control. (**j**) Western blot analysis of SORLA in scramble (siCTRL) and shSORLA (shSORLA #1, shSORLA #4) silenced MDA-MB-361 cells. α-tubulin is a loading control. (**k**) Proliferation of MDA-MB-361 shRNA cells silenced as in (**j**). Data are mean ± s.d. (n = 4 independent experiments; statistical analysis: two-way ANOVA).

IB: immunoblotting; high exp: high exposure; low exp: low exposure.

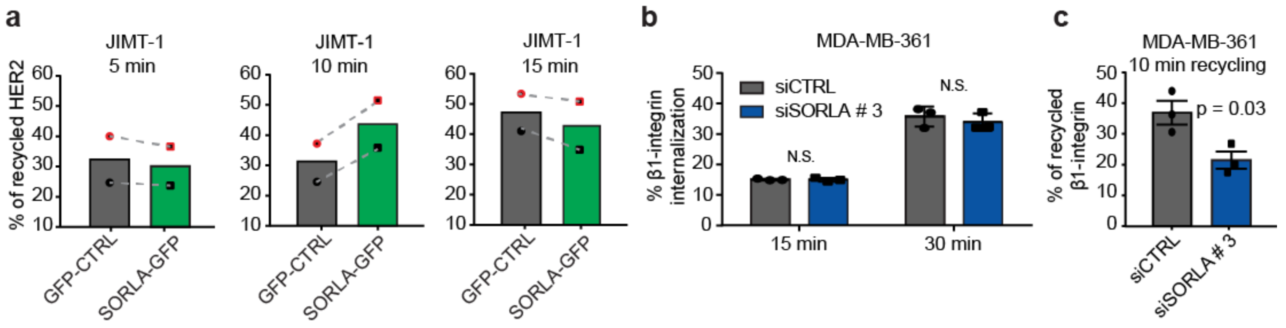

Supplemental Figure 4. (**a**) ELISA-based immunoassay analysis of biotin-labelled cell-surface HER2 recycling at 5 min, 10 min and 15 min (30 min endocytosis) in JIMT-1 cells with two different capture antibodies: red points (trastuzumab, Herceptin), black points (c-erbB-2 A0485), bars average of the two. (**b**) ELISA-based immunoassay analysis of biotin-labelled cell-surface β1 integrin (9EG7) internalization after 15 min and 30 min in MDA-MB-361 cells treated with scramble (siCTRL) and SORLA (siSORLA) siRNAs for 72 h (data are mean ± s.d.; n = 3 independent experiments; statistical analysis: unpaired Student's *t*-test). (**c**) ELISA-based immunoassay analysis of biotin-labelled cell-surface β1 integrin recycling after 10 min (30 min endocytosis) in MDA-MB-361 cells treated with scramble (siCTRL) and SORLA (siSORLA) siRNAs for 72 h (data are mean ± s.d.; n = 3 independent experiments; statistical analysis: unpaired Student's *t*-test).

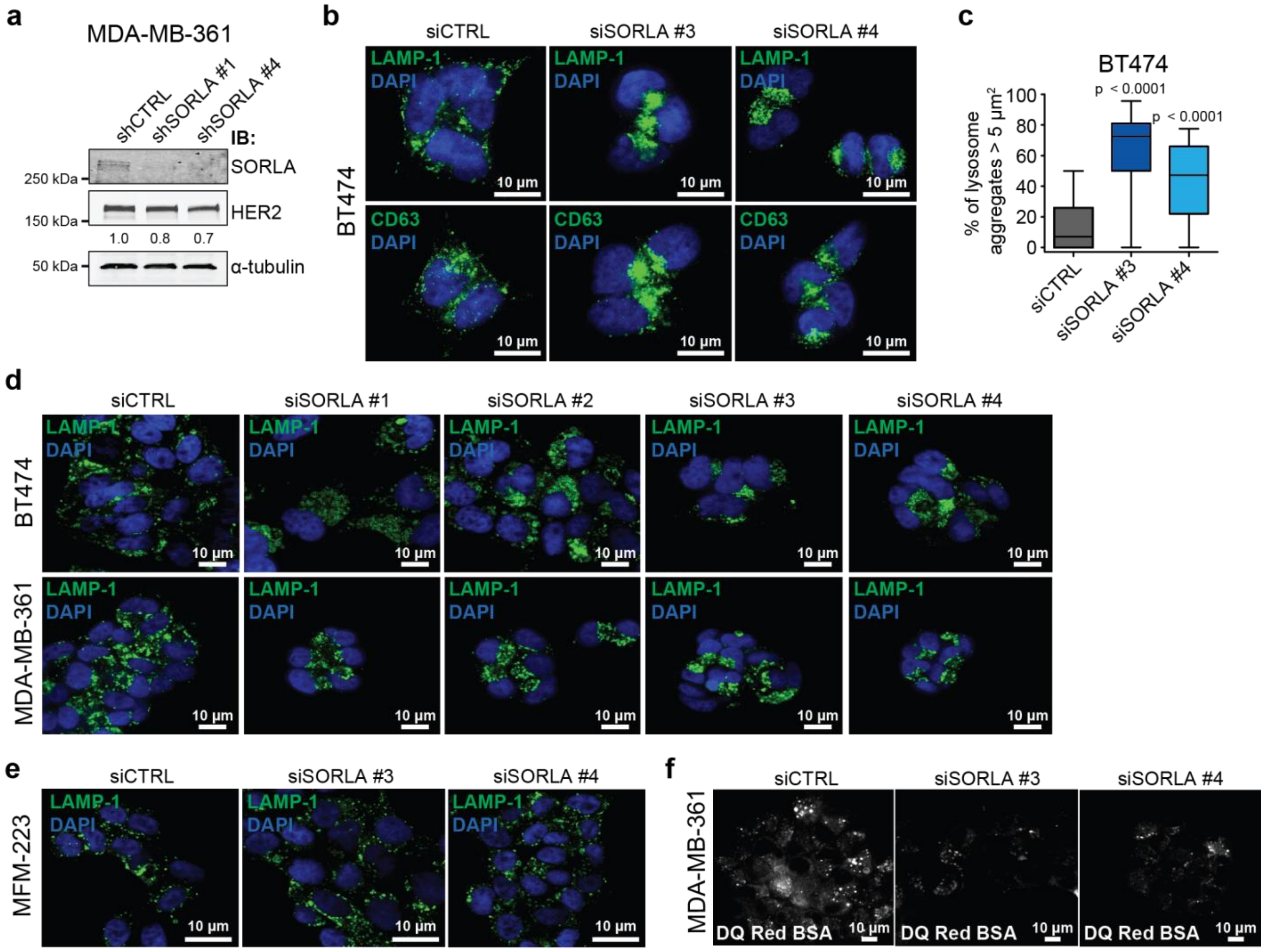

Supplementary Figure 5. (**a**) Western blot analysis of SORLA and HER2 levels in scramble (shCTRL) and shSORLA (shSORLA #1, shSORLA #4) silenced MDA-MB-361 cells. α-tubulin is a loading control. Numbers are HER2 levels relative to CTRL. (**b**) Confocal microscopy images of LAMP1, CD63 and DAPI (for nucleus) in scramble (siCTRL) and siSORLA (siSORLA#3, siSORLA#4) silenced BT474 cells. (**c**) Quantification of late-endosomes/lysosome aggregation after SORLA silencing in BT474 cells (performed only from LAMP1 staining). LAMP1-positive structures ≥ 5 μm^2 were considered as lysosome aggregates. Data are displayed as box plots (n = 32 siCTRL, 35 siSORLA #3 and 24 siSORLA #4 cells, three independent experiments; statistical analysis: Mann-Whitney test). (**d**) Confocal microscopy images of LAMP1 and DAPI (for nucleus) in scramble (siCTRL) and siSORLA (siSORLA #1, siSORLA #2, siSORLA#3, siSORLA#4) silenced BT474 and MDA-MB-361 cells. (**e**) Confocal microscopy images of LAMP1 and DAPI in scramble (siCTRL) and siSORLA (siSORLA#3, siSORLA#4) silenced non-HER2-amplified MFM-223 breast cancer cells. (**f**) Microscopy images of DQ Red BSA degradation (loss of quenching) in 48 h BSA-loaded MDA-MB-361 cells after scramble (siCTRL) or SORLA (siSORLA #3 and siSORLA #4) silencing.

a

Table I. SORLA and EGFR expression correlation in bladder cancer TMA

	EGFR (0)	EGFR (1)	EGFR (2)	EGFR (3)
SORLA (0)	19 (42 %)	17 (38 %)	8 (18 %)	1 (2 %)
SORLA (1)	10 (20 %)	27 (53 %)	13 (25 %)	1 (2 %)
SORLA (2)	14 (22 %)	32 (51 %)	16 (25 %)	1 (2 %)

Chi-square test, p = 0.226

b

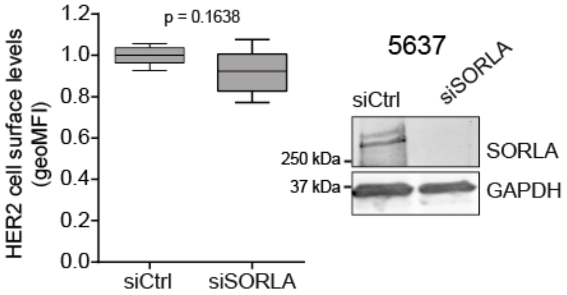

c

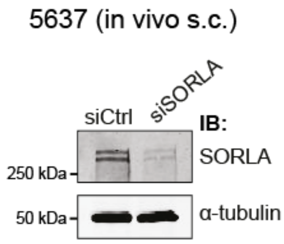

d

Table II. TCGA analysis of correlation between SORLA and HER2 in different cancer types

Cancer type	Pearson correlation coefficient	P-value
Testicular germ cell tumors	0.442	8.68E - 08
Cervical squamous cell carcinoma and endocervical adenocarcinoma	0.139	0.0155
Kidney Renal clear cell carcinoma	0.322	2.67E - 14
Sarcoma	0.352	5.83E - 09
Thymoma	0.587	1.78E - 12

Supplementary Figure 6. (**a**) Correlation analyses of SORLA and EGFR expression in bladder cancer TMA material. Numbers indicate staining intensity, 0=negative. (**b**) Flow cytometry analysis of HER2 levels on the cell surface in 5637 cells after silencing with control (siCTRL) and SORLA (siSORLA) siRNAs (mean ± s.d. of n = 3 independent experiments; statistical analysis: unpaired Student's *t*-test). (**c**) Western blot analysis of SORLA levels in scramble (siCTRL) and siSORLA (siSORLA#3) silenced 5637 bladder cancer cells used in the *in vivo* xenograft assay in Fig. 6d, e. α-tubulin is a loading control. (**d**) Correlation between SORLA and HER2 expression in different neoplasms based on TCGA data.

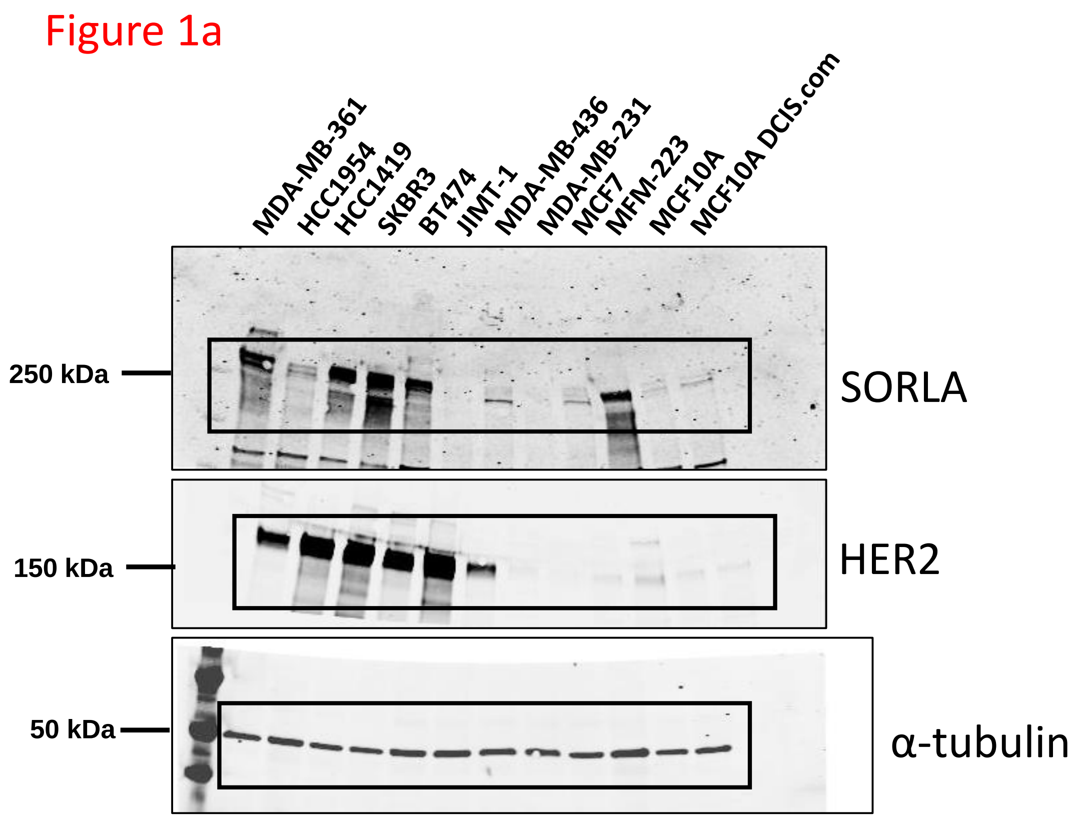

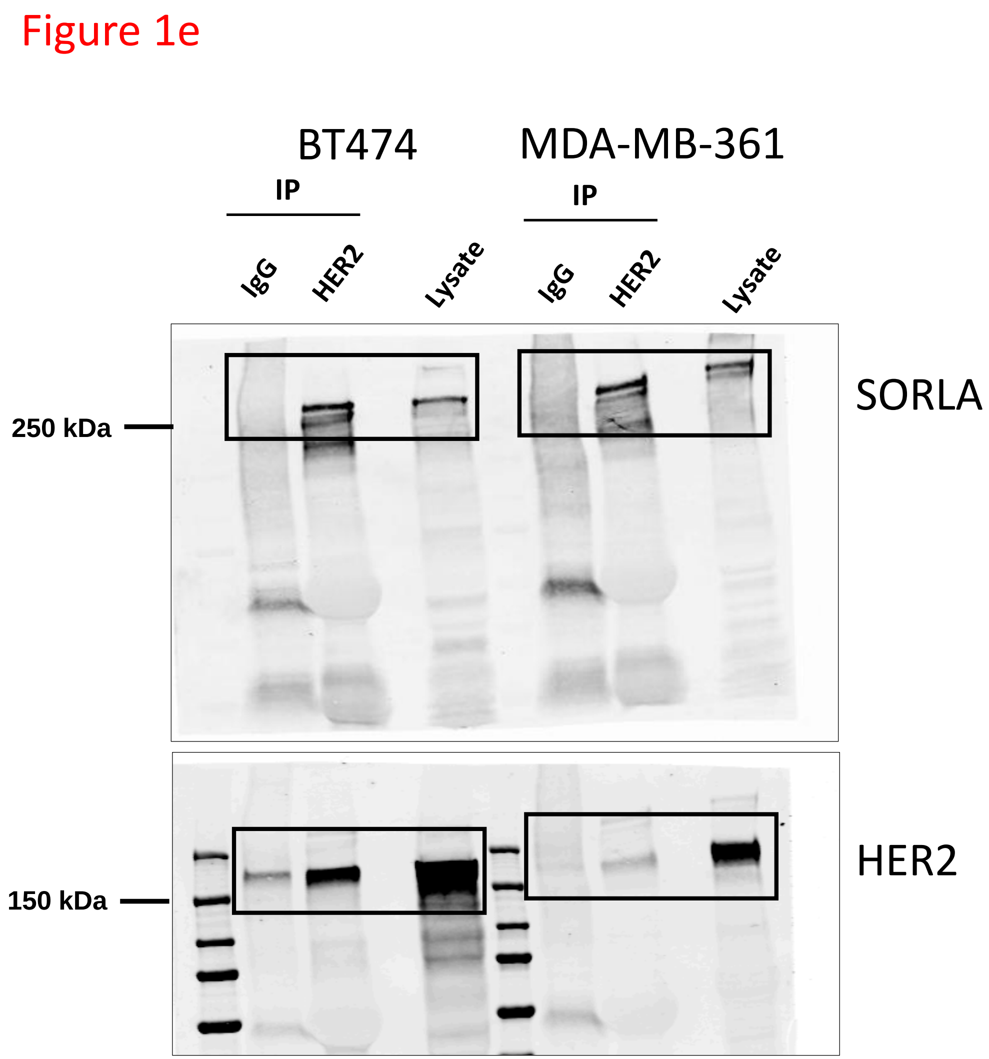

Supplementary Figure 7. Original western blots of the manuscript (1 of 12)

Figure 1g

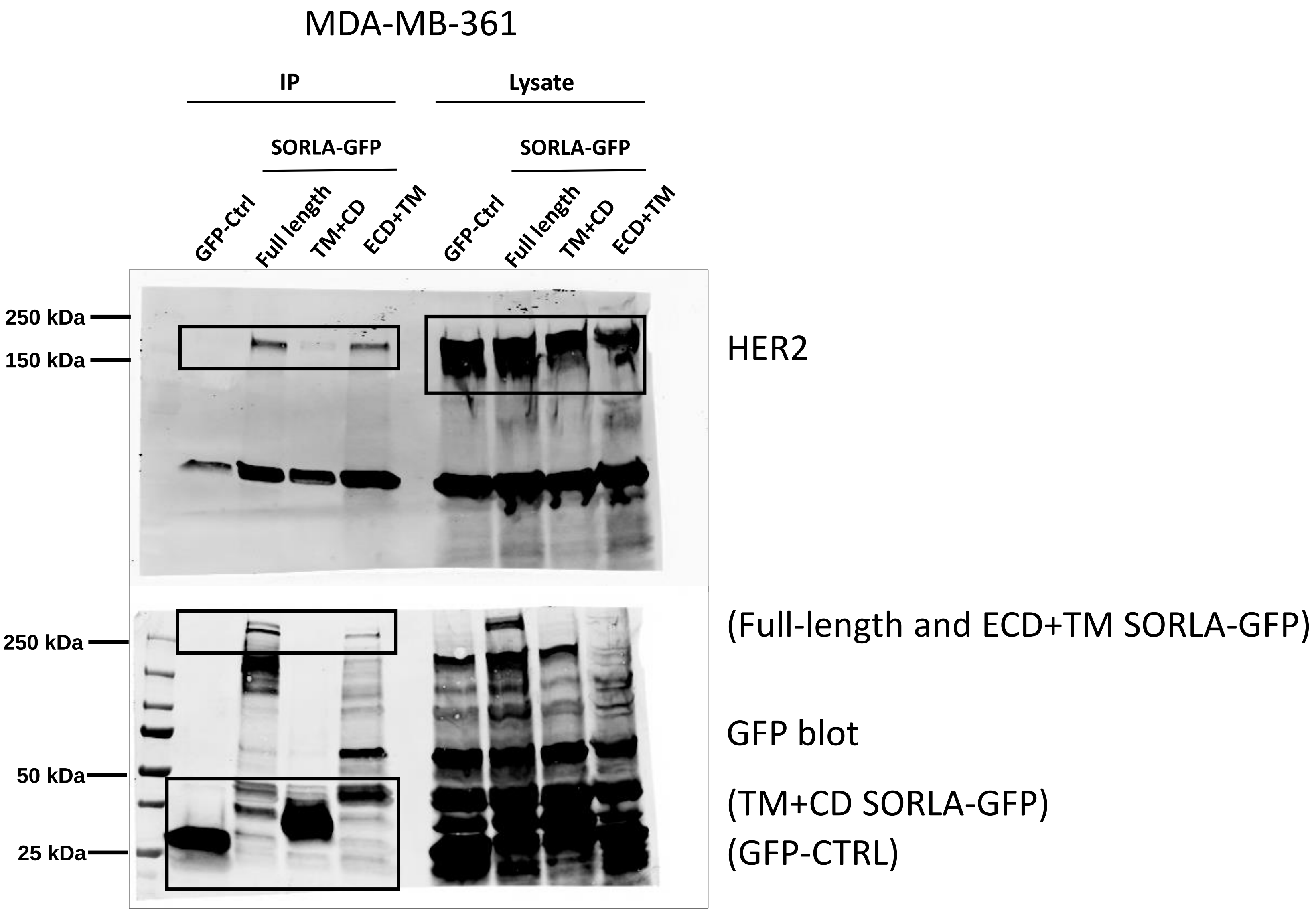

Supplementary Figure 7. Original western blots of the manuscript (2 of 12)

Figure 2c

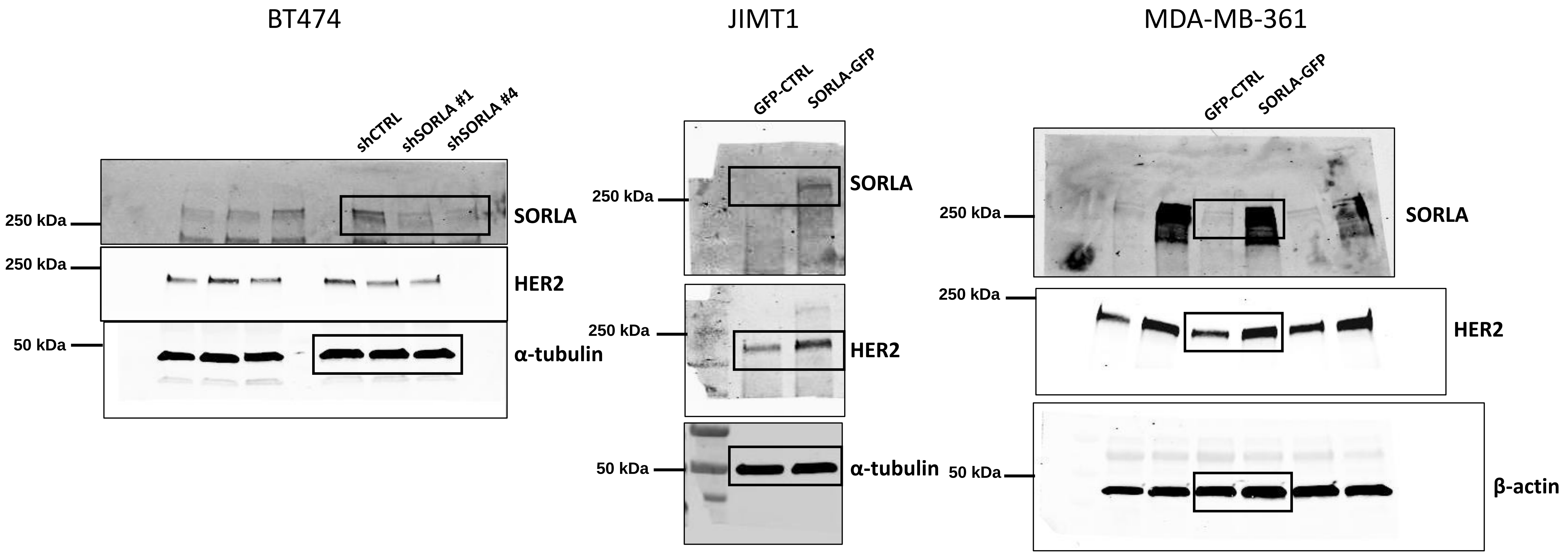

Supplementary Figure 7. Original western blots of the manuscript (3 of 12)

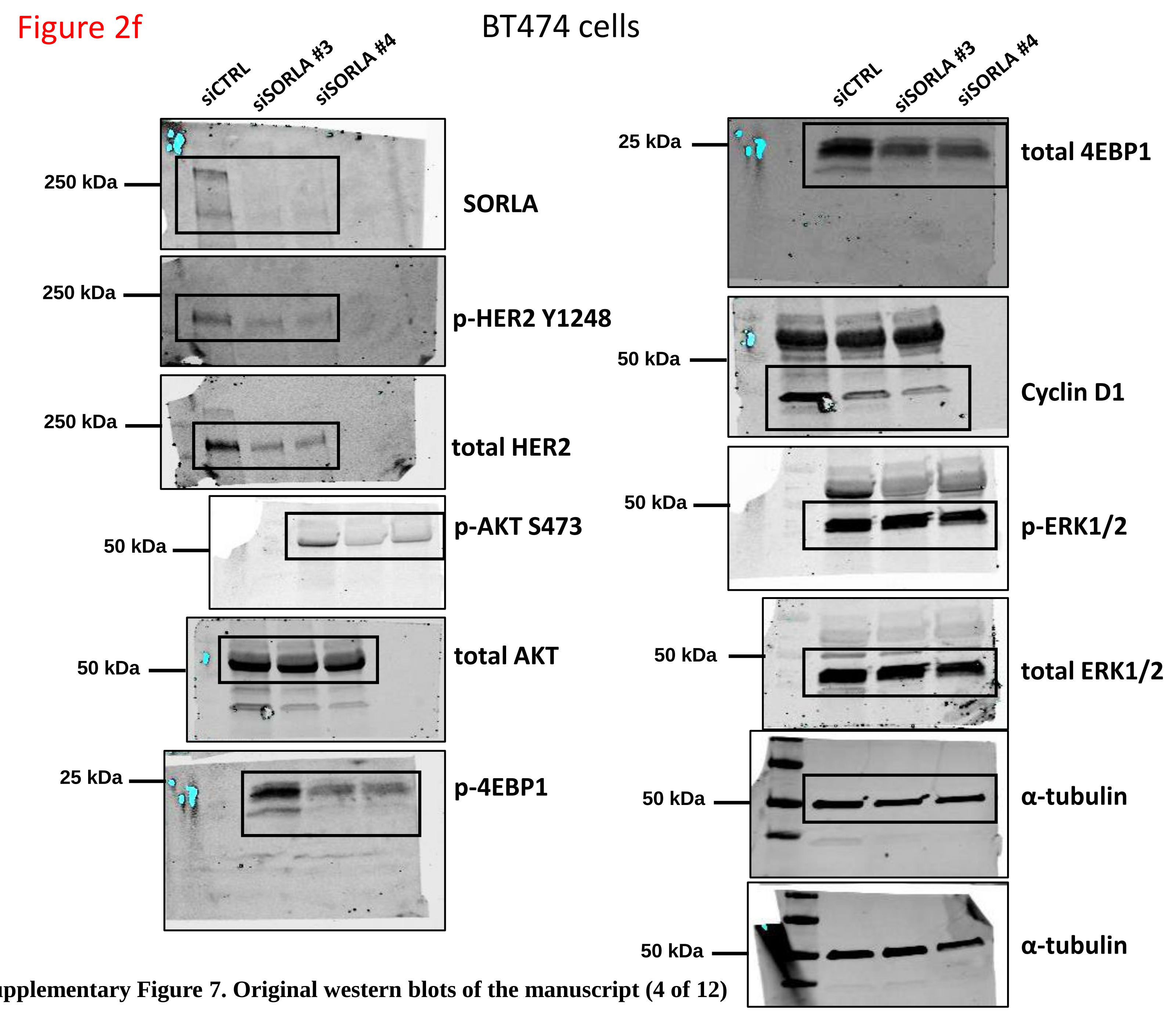

Supplementary Figure 7. Original western blots of the manuscript (4 of 12)

Figure 3h

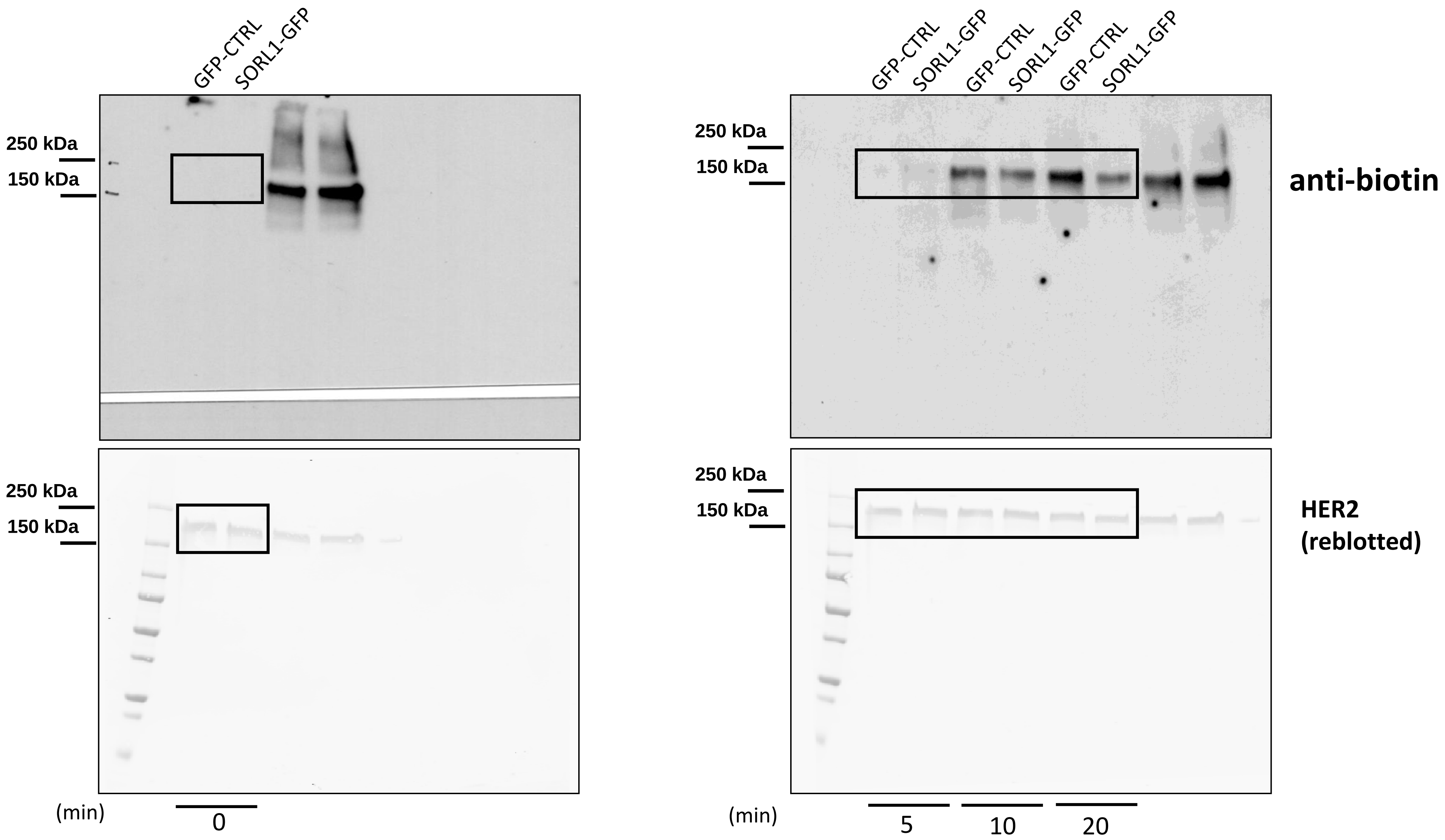

Supplementary Figure 7. Original western blots of the manuscript (5 of 12)

Figure 4h

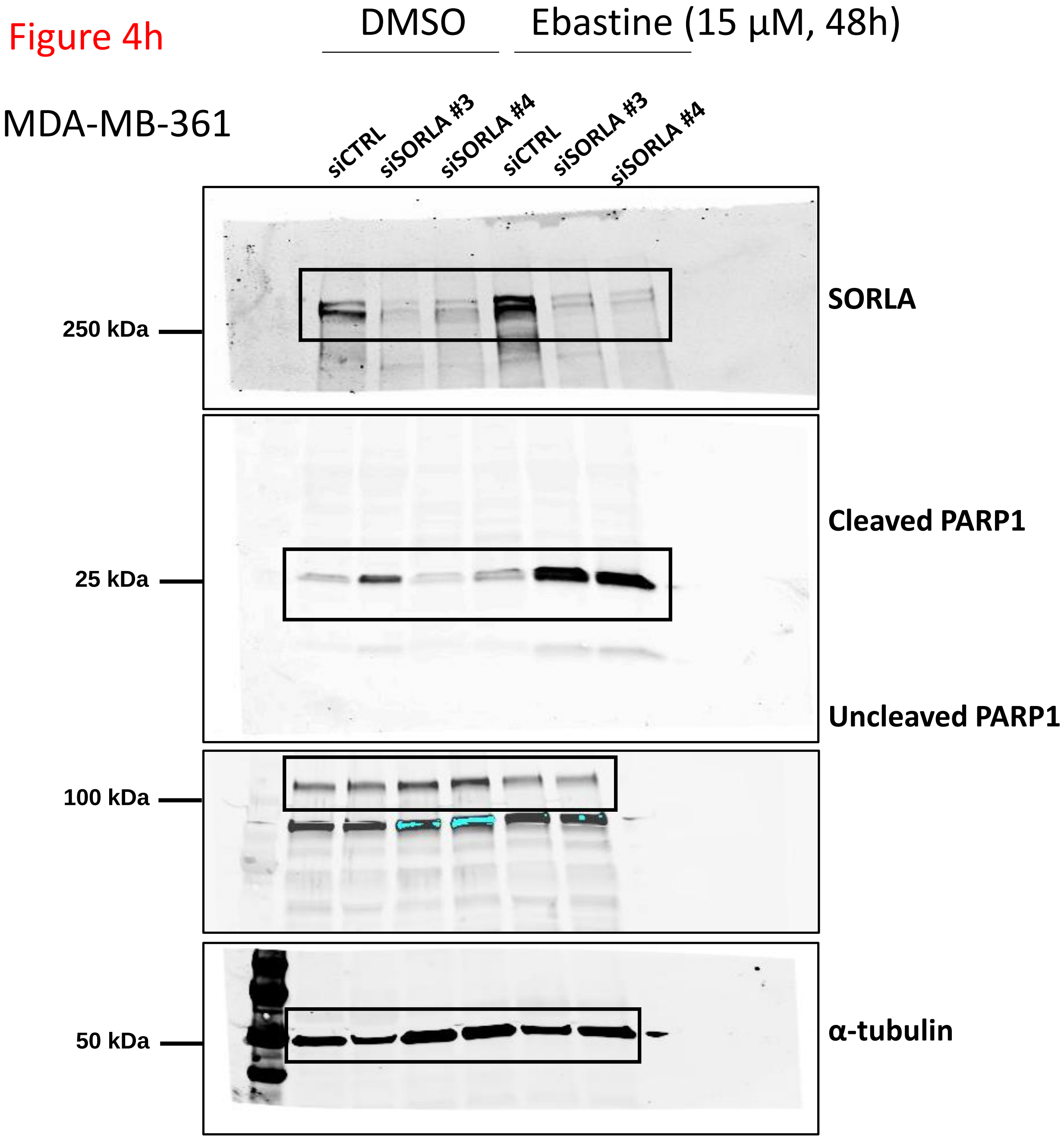

Figure 6b

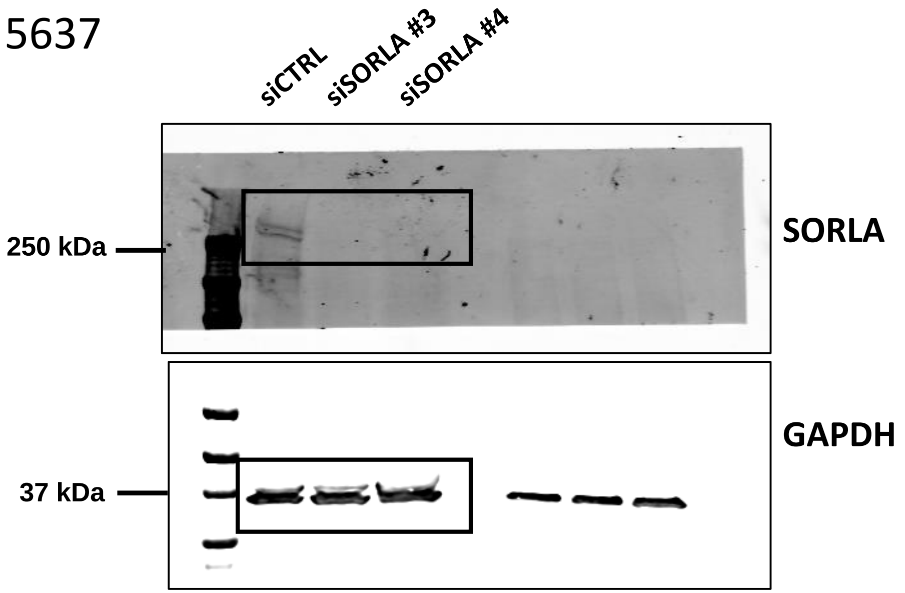

Supplementary Figure 7. Original western blots of the manuscript (6 of 12)

Supplemental figure 1a

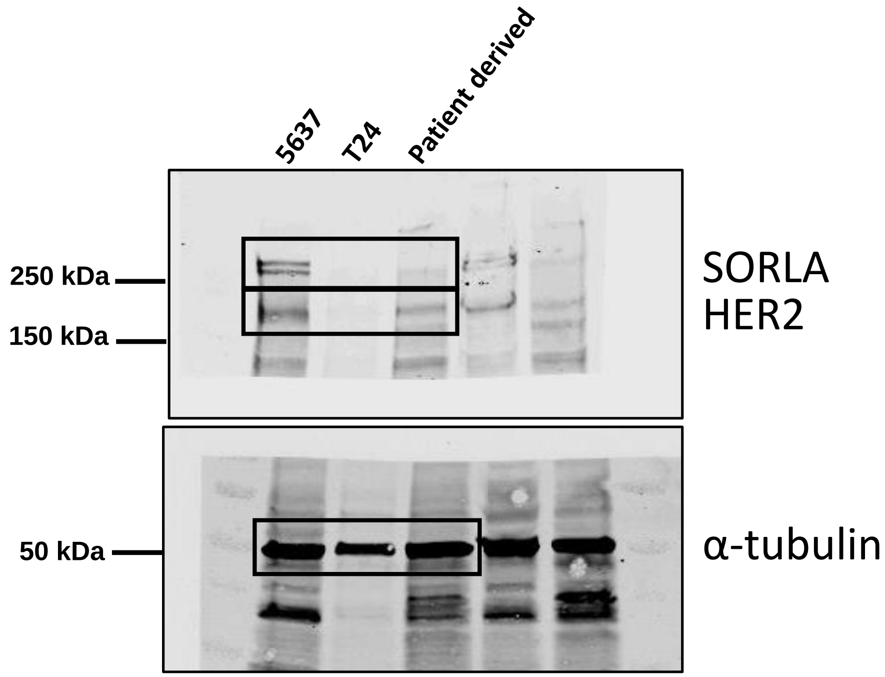

Supplemental figure 1b

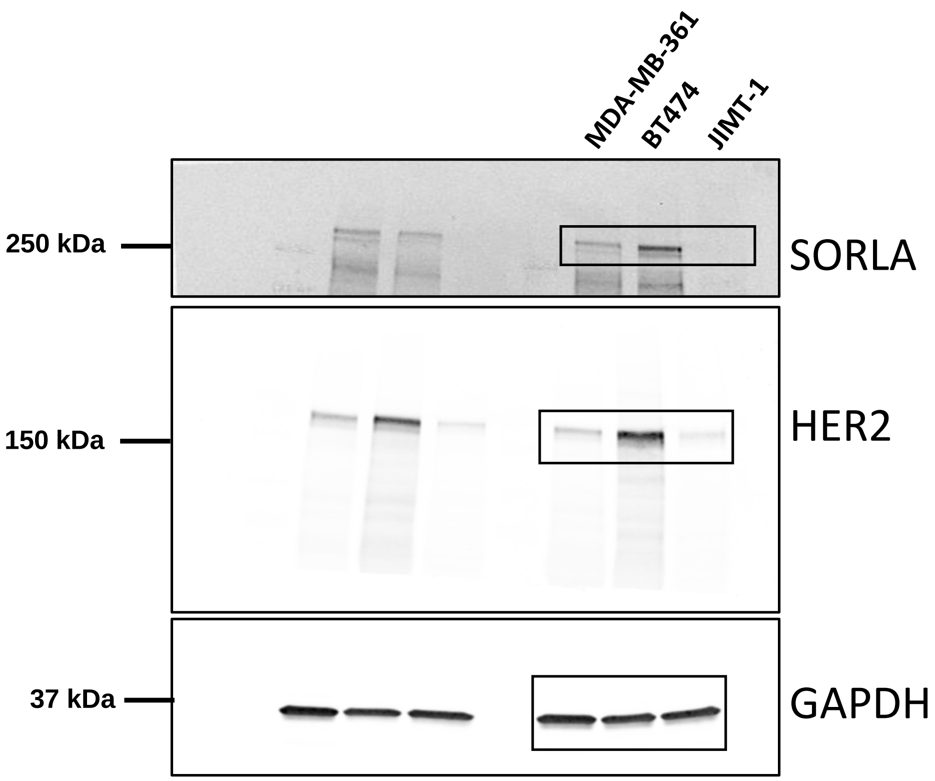

Supplementary Figure 7. Original western blots of the manuscript (7 of 12)

Supplementary Figure 2c

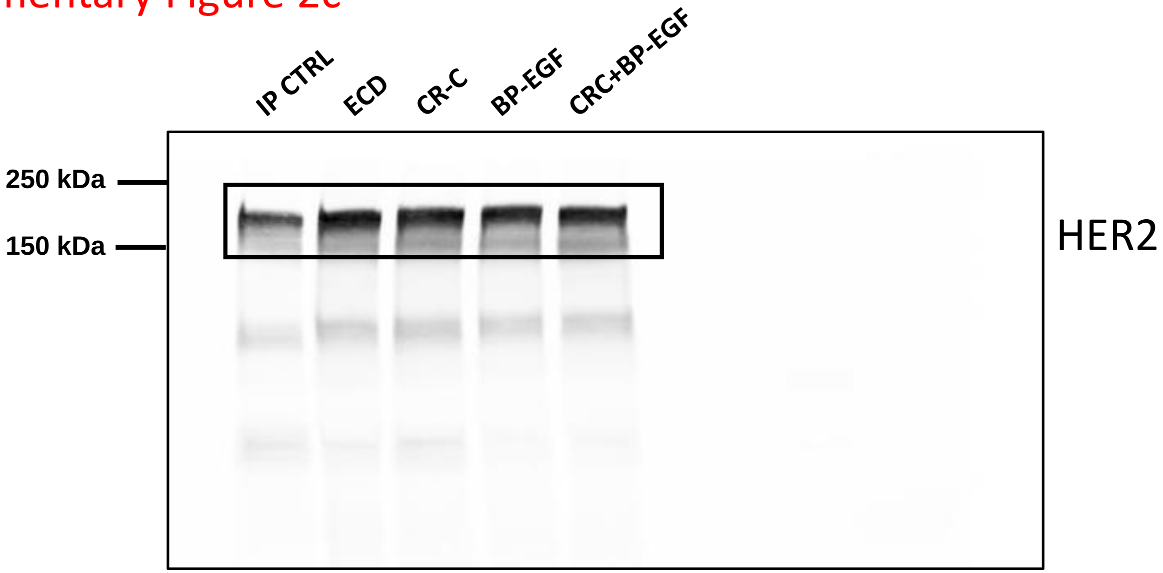

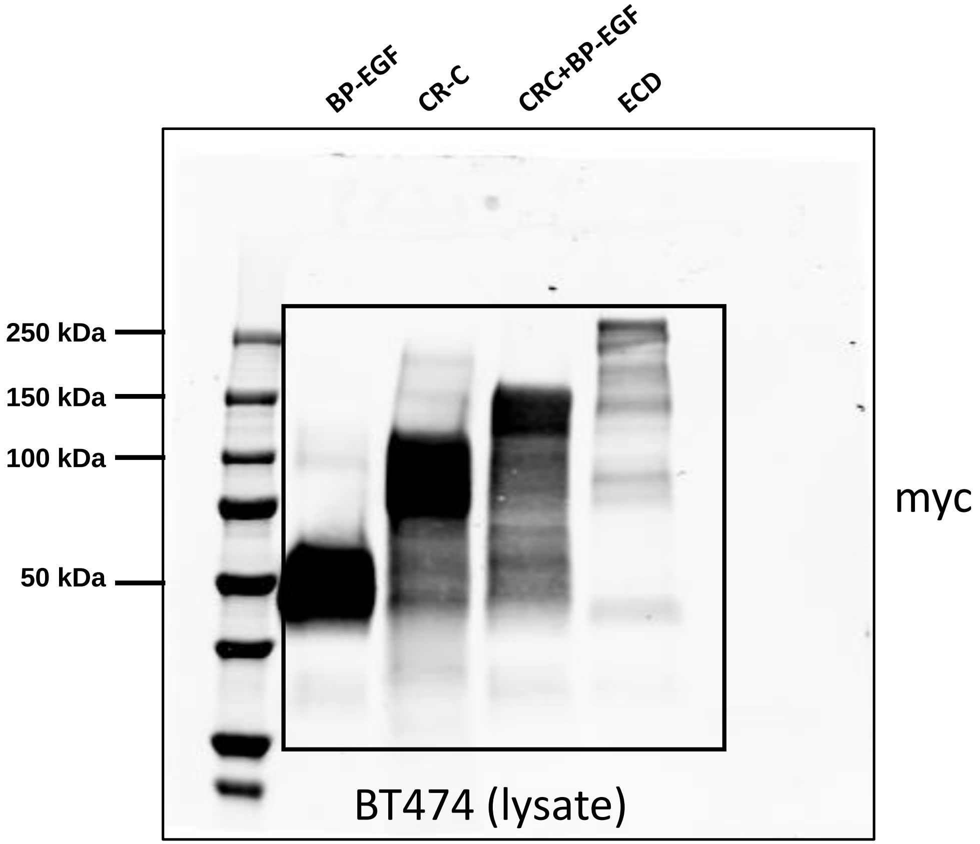

Supplementary Figure 7. Original western blots of the manuscript (8 of 12)

Supplementary Figure 3c and 3g

MFM-223 (3g) BT474 (3c)

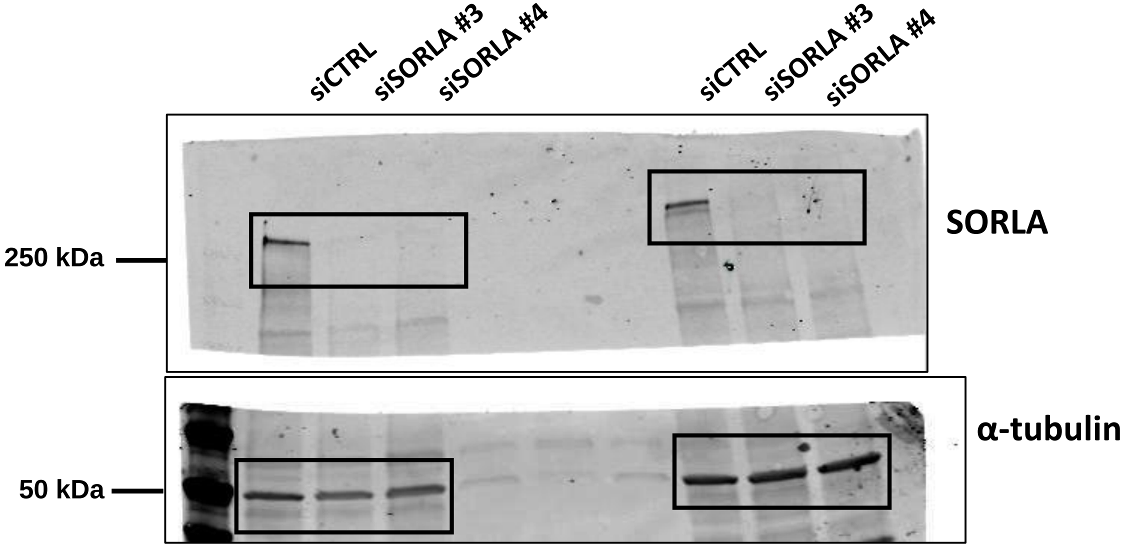

Supplementary Figure 3d

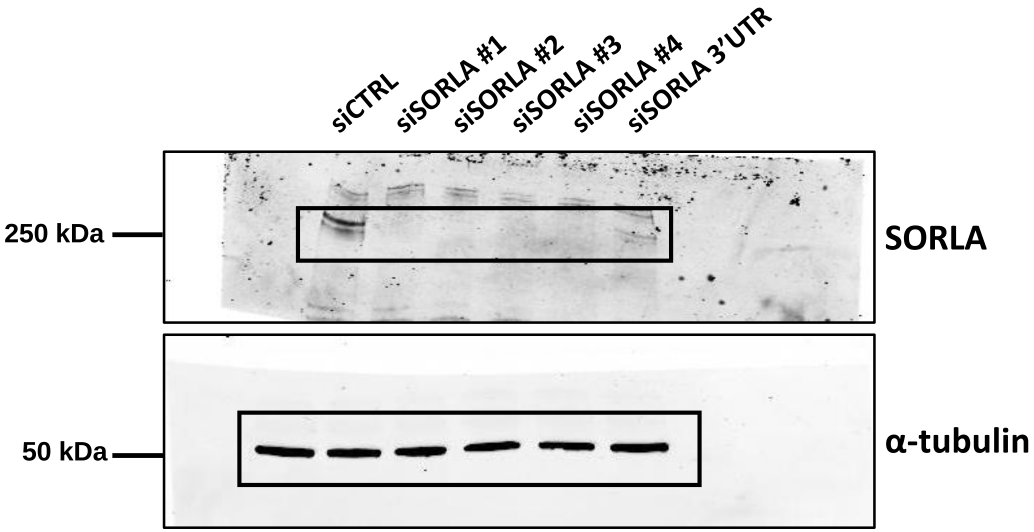

Supplementary Figure 3f

JIMT-1

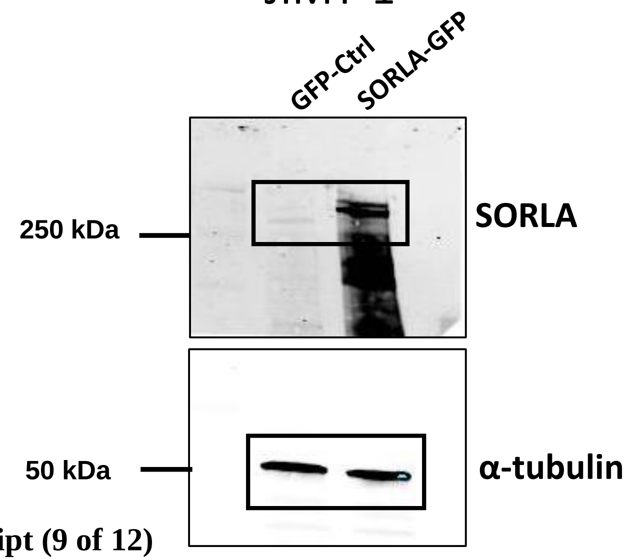

Supplementary Figure 7. Original western blots of the manuscript (9 of 12)

Supplementary Figure 3h

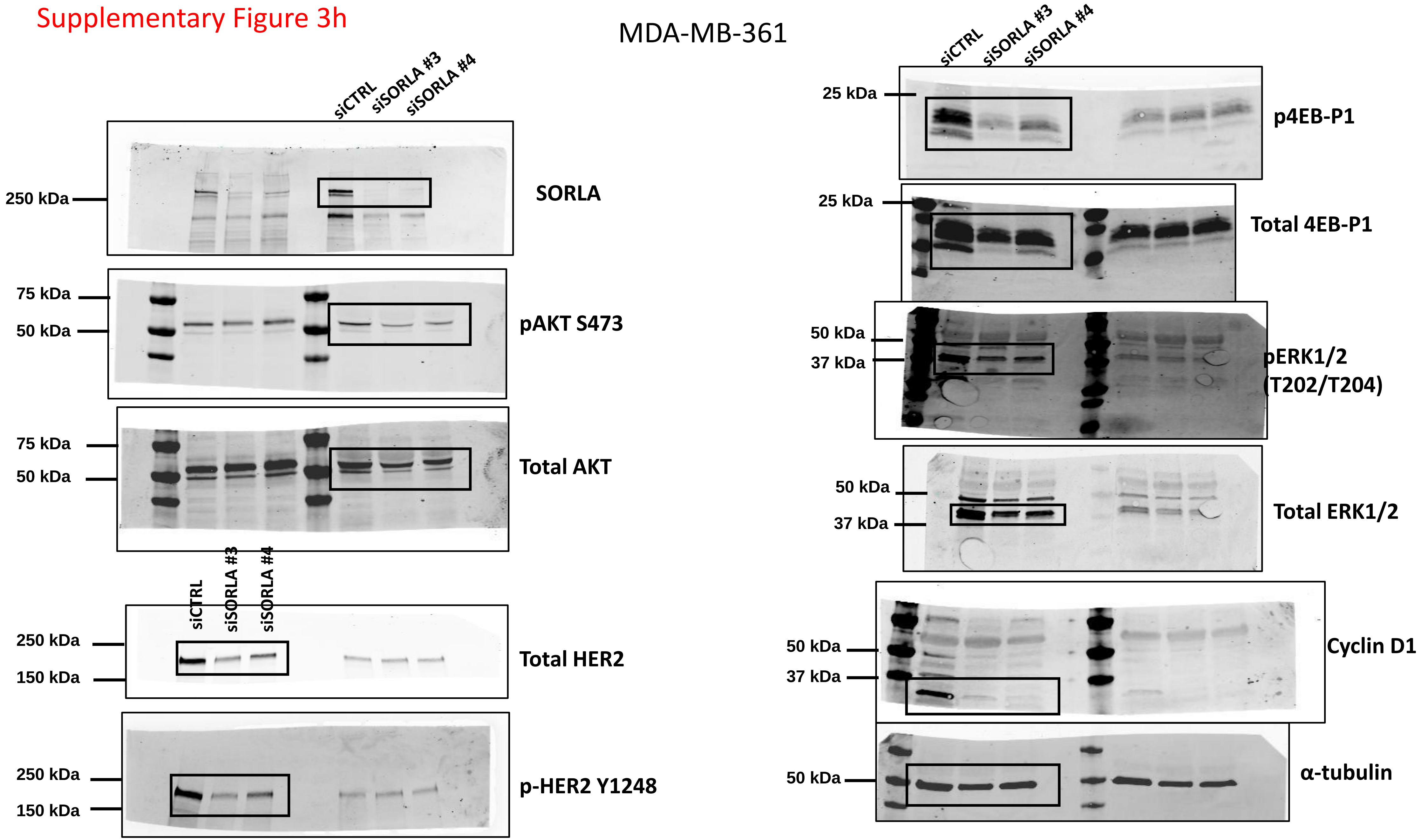

Supplementary Figure 7. Original western blots of the manuscript (10 of 12)

Supplementary Figure 3i

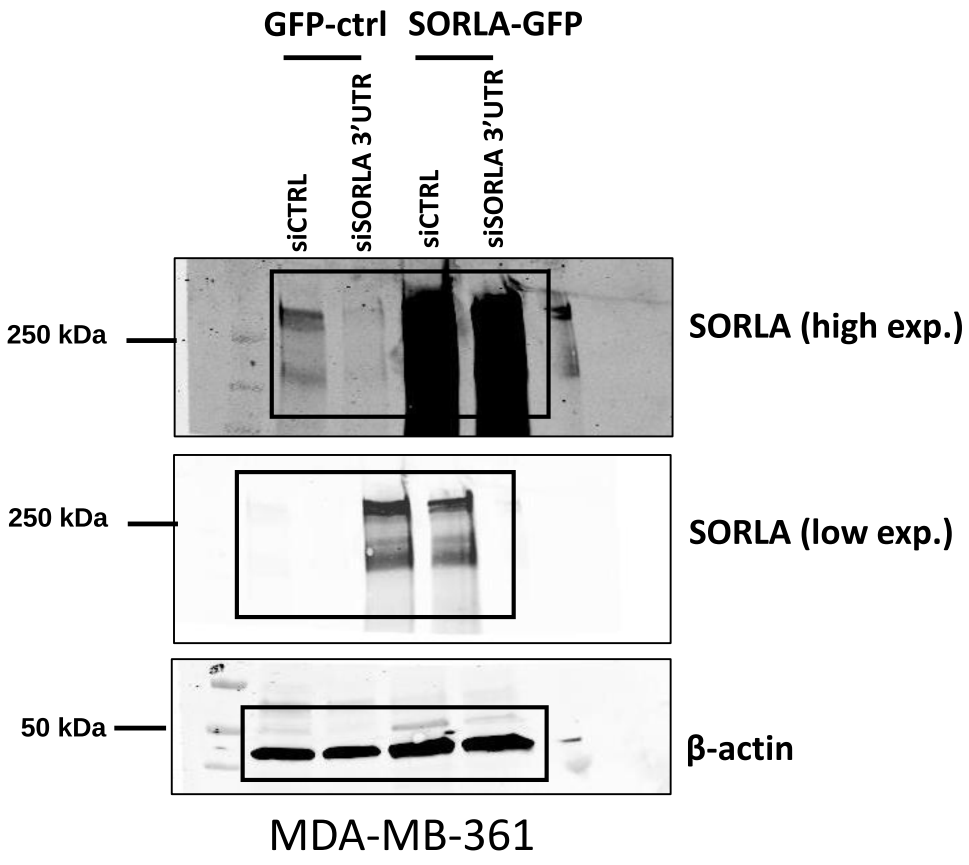

Supplementary Figure 7. Original western blots of the manuscript (11 of 12)

Supplementary Figure 5a

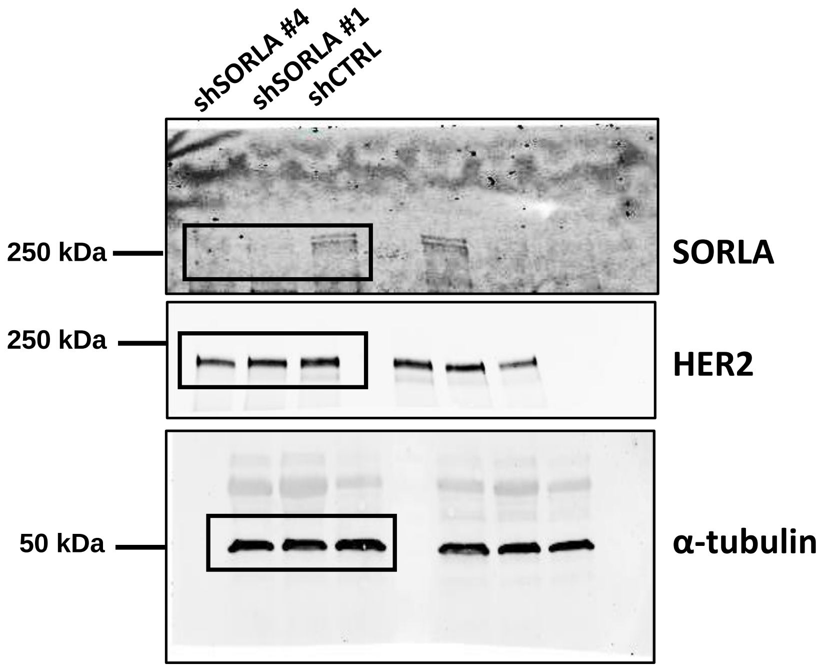

Supplementary Figure 6b

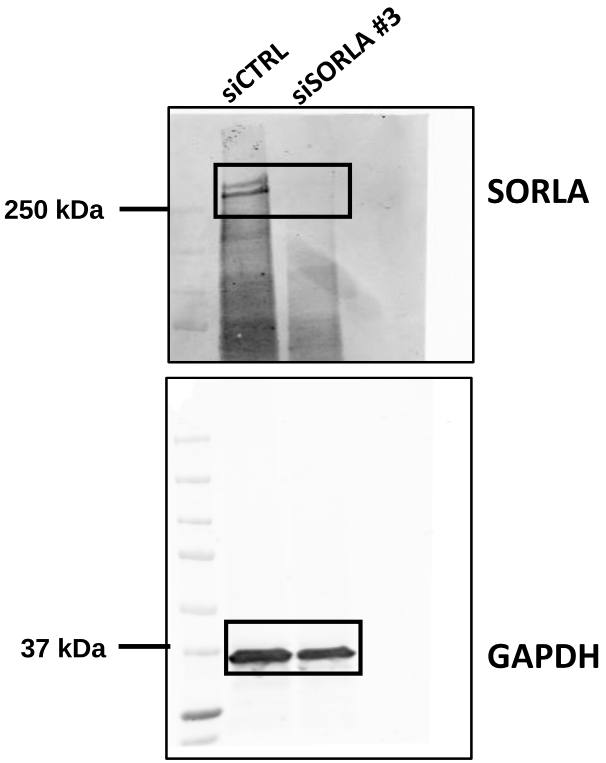

Supplementary Figure 6c

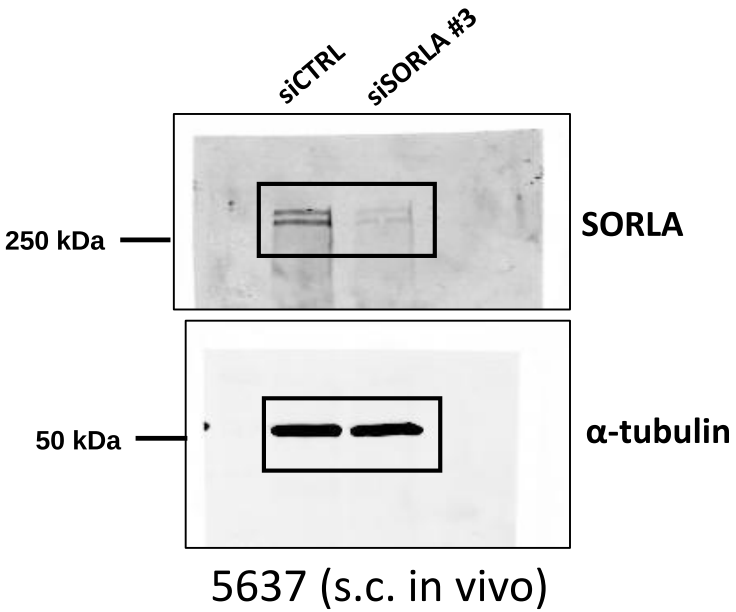

Supplementary Figure 7. Original western blots of the manuscript (12 of 12)

Figure 1b and Supplementary Figure 1e

MDA-MB-361 cells

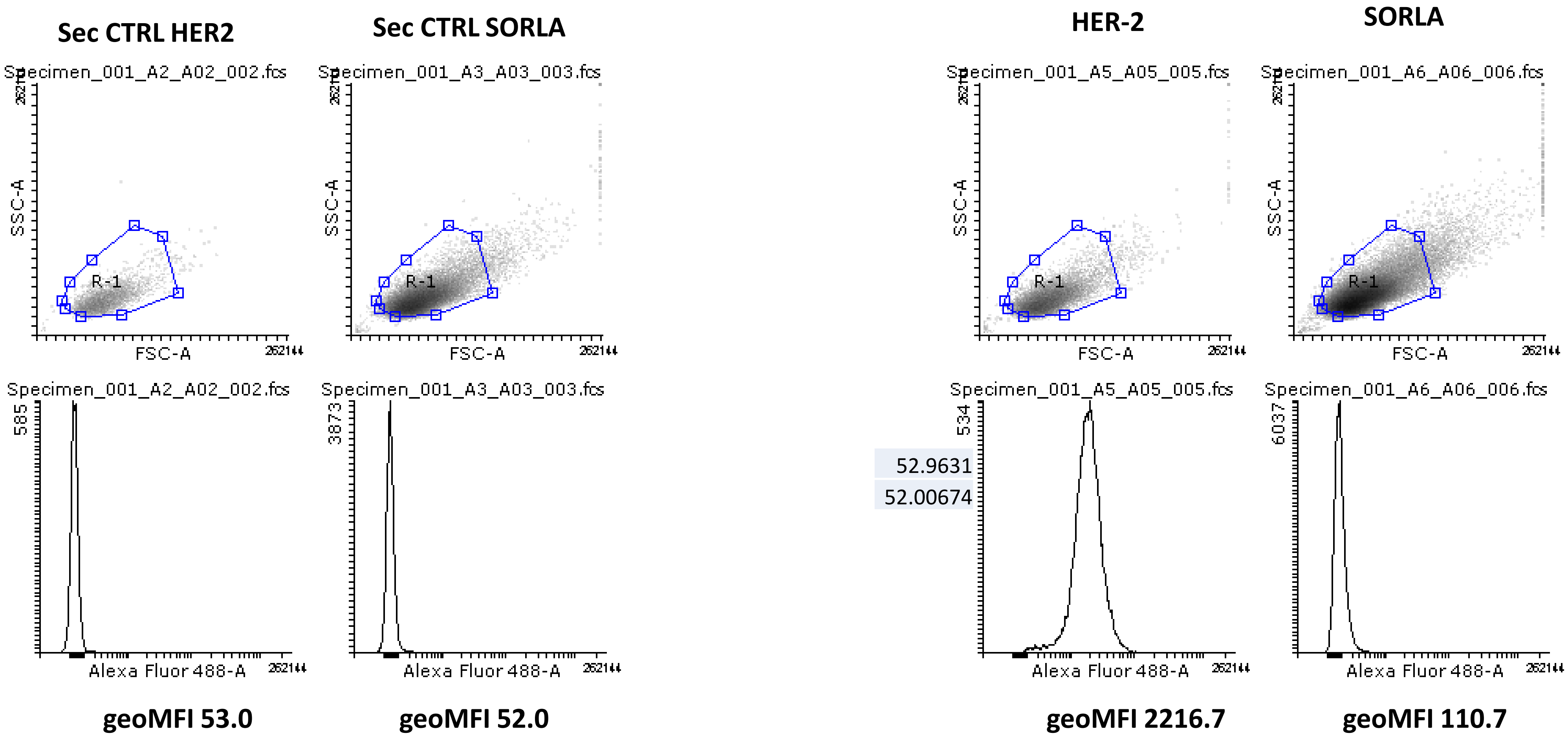

Supplementary Figure 8. Representative raw flow cytometry data and gating (1 of 7)

Figure 1b and Supplementary Figure 1e

BT474 cells

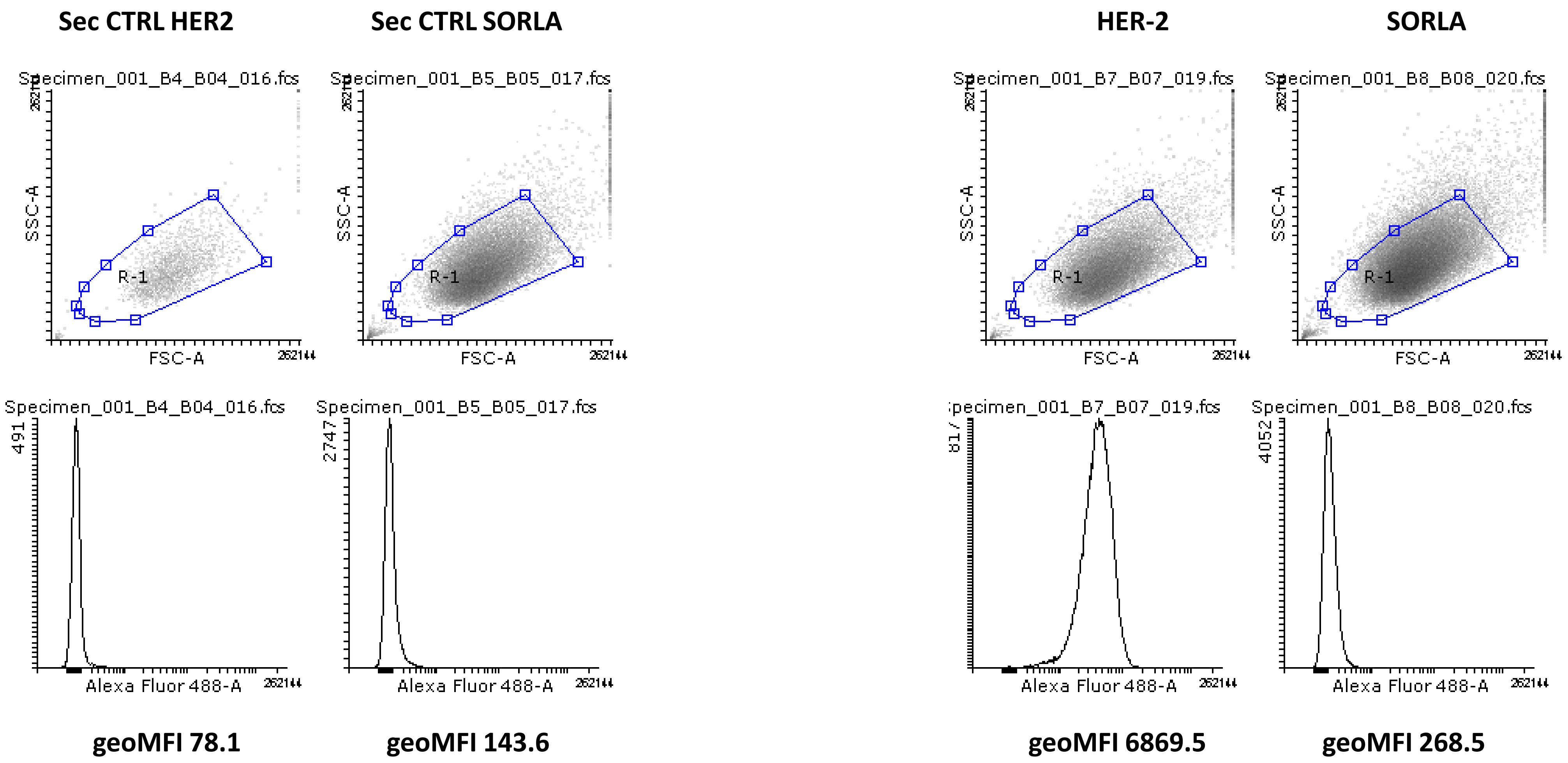

Supplementary Figure 8. Representative raw flow cytometry data and gating (2 of 7)

Figure 1b and Supplementary Figure 1e

JIMT-1 cells

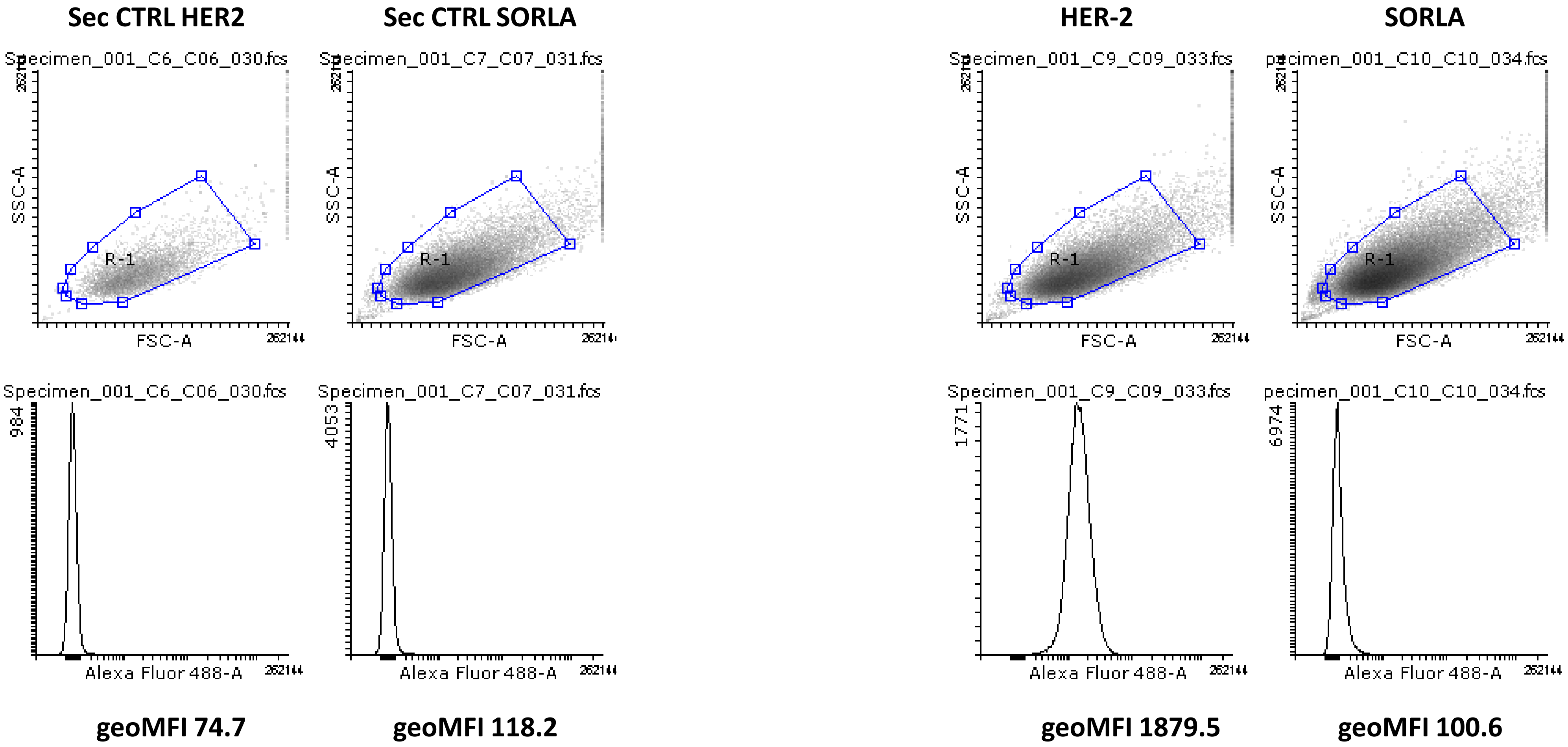

Supplementary Figure 8. Representative raw flow cytometry data and gating (3 of 7)

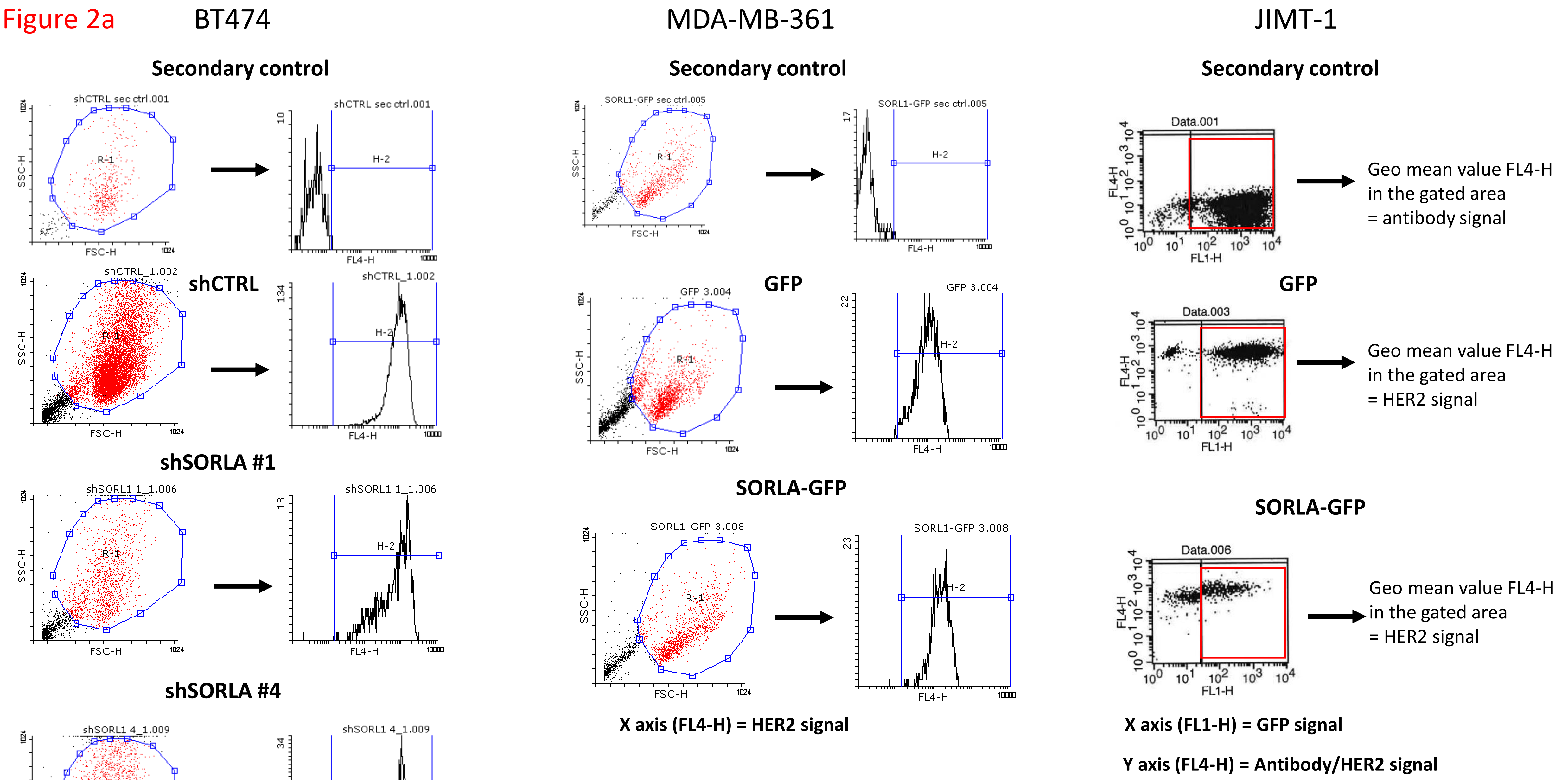

Supplementary Figure 8. Representative raw flow cytometry data and gating (4 of 7)

Figure 4f

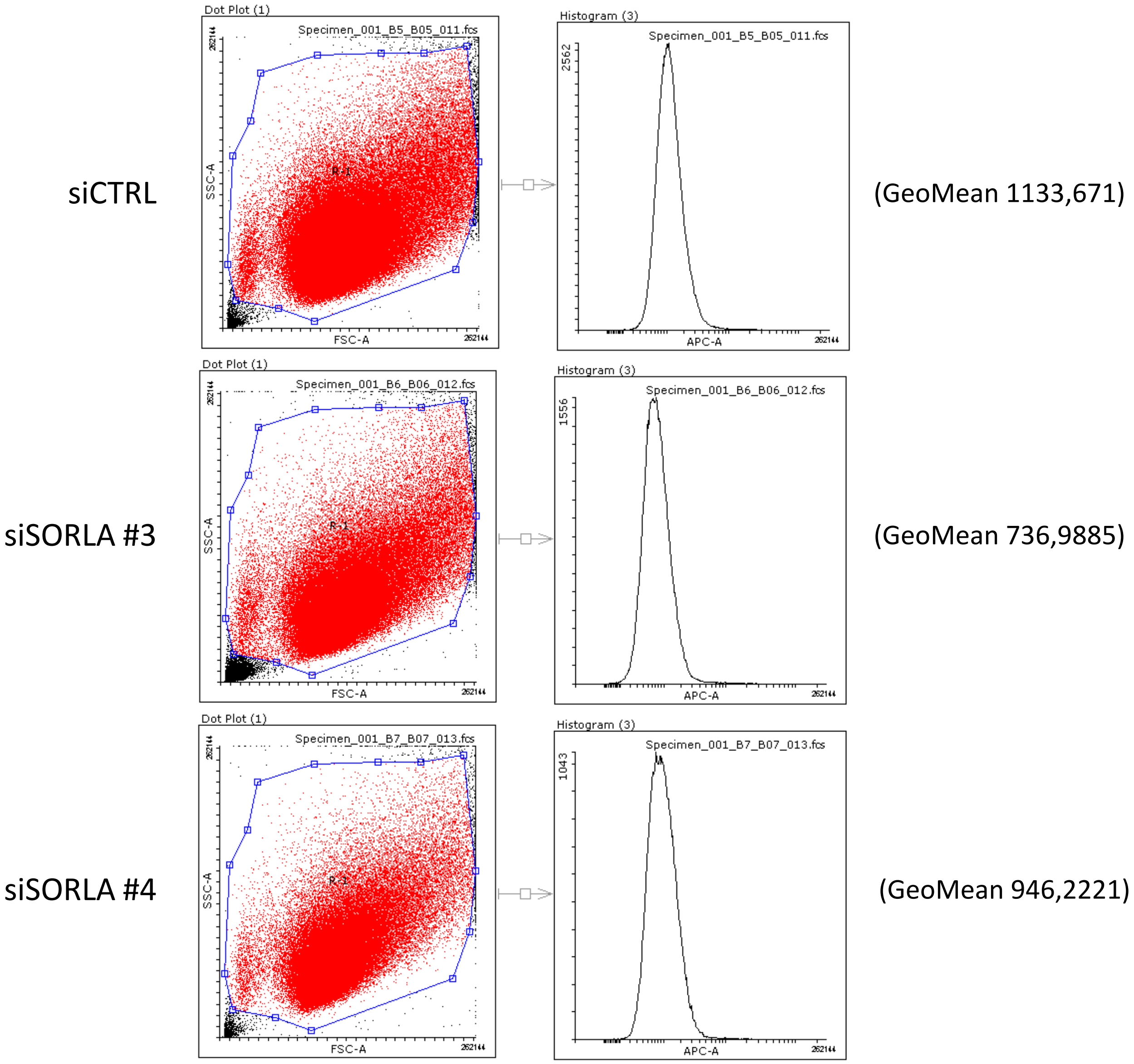

Supplementary Figure 8. Representative raw flow cytometry data and gating (5 of 7)

Supplementary Figure 3b

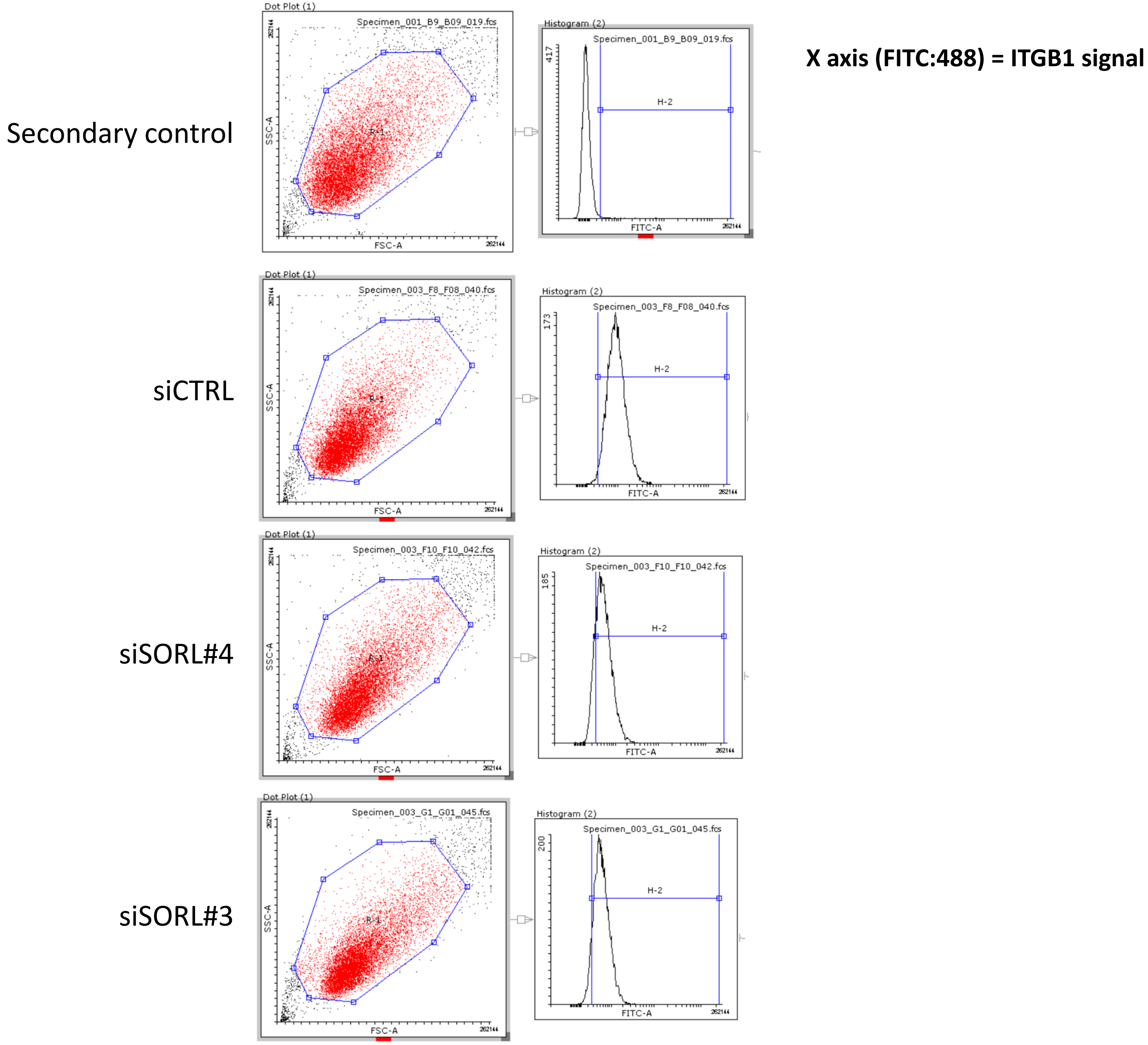

Supplementary Figure 8. Representative raw flow cytometry data and gating (6 of 7)

Supplementary Figure 6b

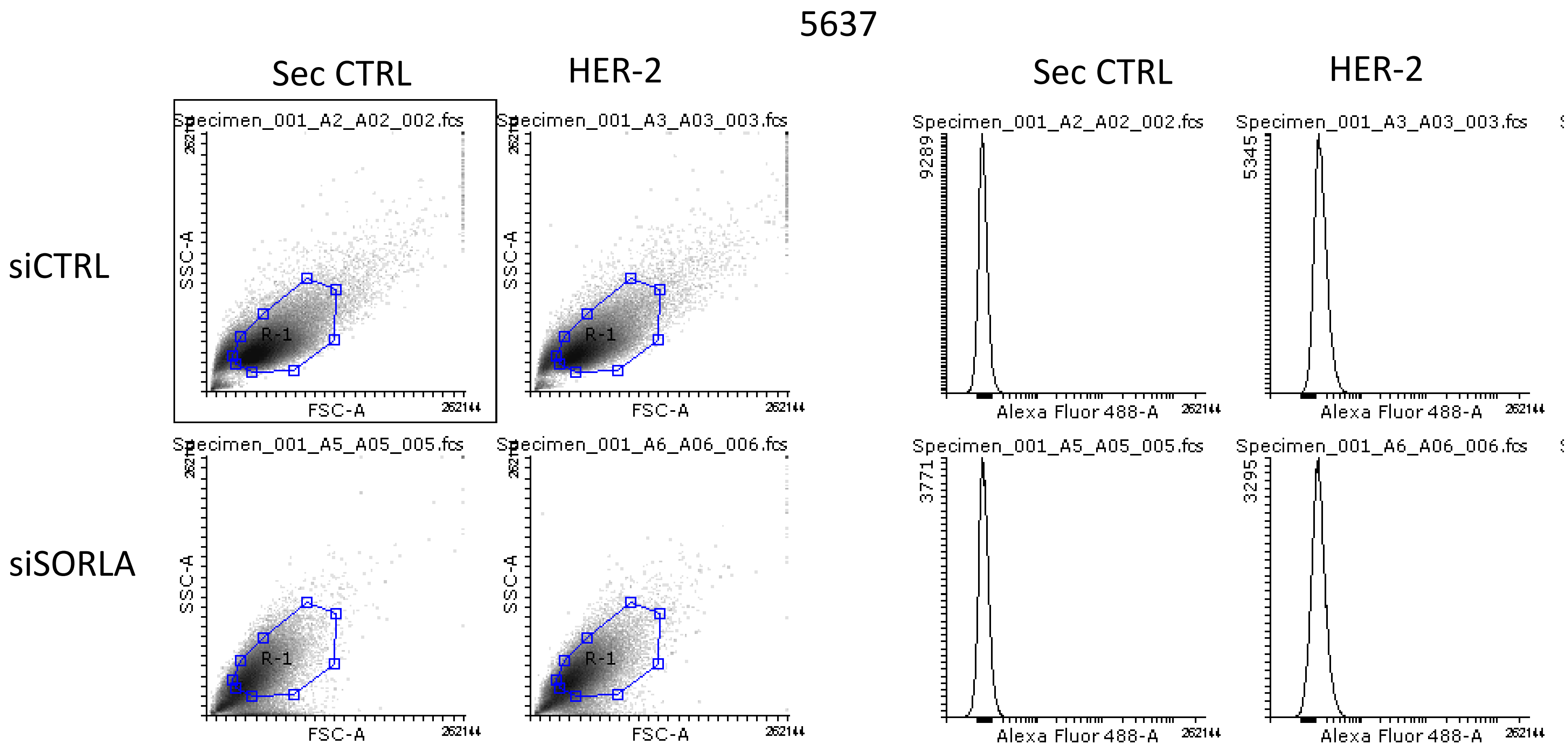

Supplementary Figure 8. Representative raw flow cytometry data and gating (7 of 7)

Antibody	Manufacturer	Catalogue No.	Application	Dilution
HER2/ErbB2 (e2-4001 + 3B5)	Thermo Scientific	MA5-14057	WB<br>IP	1:1000<br>2 µg/ml
ErbB2 (9G6)	Abcam	Ab16899	FACS	1:300
HER2/neu	Ventana	clone 4B5	IHC	Ready to use (6 µg/ml)
EGFR	Ventana	Clone 5B7	IHC	Ready to use (0.4 µg/ml)
SORLA	C.M. Petersen (Aarhus U)		IF	1:200
SORLA	J. Gliemann (Aarhus U)	Goat polyclonal	IP/FACS	1 µg/1:200
SORL1 (histological staining)	Atlas Antibodies	HPA031321	IHC	1:300
LR11 (SORL1)	BD Transduction Lab	612633	WB	1:1000
p-AKT (S473)	CST	#9271S	WB	1:1000
p-AKT (T308)	CST	9275S	WB	1:1000
Total AKT	CST	#9272	WB	1:1000
LAMP-1	Santa Cruz	SC-20011 (H4A3)	IF	1:50
EEA-1	Santa Cruz	sc-6415	IF	1:50
VPS35	Abcam	ab10099	IF	1:250
p4E-BP1 (T37/46)	CST	2855S	WB	1:1000
Total 4E-BP1	CST	9452S	WB	1:1000
phospho-ERK p-p44/42 MAPK T202/Y204	CST	4370S	WB	1:1000
Total ERK p44/p42 MAPK	CST	9102S	WB	1:1000

GAPDH	HyTest	5G4MaB6C5	WB	1:500
α-tubulin	Hybridoma bank	Identifier: 12g10<br>anti-alpha-tubulin	WB	1:1000
Histone H3	CST	4499	WB	1:2000
β-actin	Sigma	A1978	WB	1:2500
CD63	Hybridoma Bank	H5C6	IF	1:300
Cleaved PARP1 (E51)	Abcam	Ab32064	WB	1:1000
Cyclin D1 (72-13G)	Santa cruz	SC-450	WB	1:1000
Rab11 (D4F5)	CST	#5589	IF	1:250
PARP1 (H-250)	Santa Cruz	SC-7150	WB	1:500
Rab5 (C8B1)	CST	#3547	IF	1:500
(Her2) Trastuzumab	Roche	CAS-180288-69-1	Live-cell imaging/IF<br>ELISA	1:200 from 30ug/ml stock<br>5 µg/ml
c-erbB-2	Dako	A0485	ELISA	5 µg/ml
9EG7 (β1-integrin)	BD	553715	ELISA	5 µg/ml

Supplementary Table 1. Antibodies and dilutions used in this study.

Oligonucleotide	SOURCE	Catalogue No.
ON-TARGETplus siRNA against SORLA #1 (CCAUGAAUAUCACAGCUUA)	Dharmacon	J-004722-08
ON-TARGETplus siRNA against SORLA #2 (GACAGGAGCUACAAAGUAA)	Dharmacon	J-004722-06
ON-TARGETplus siRNA against SORLA #3 (CCGAAGAGCUUGACUACUU)	Dharmacon	J-004722-07
ON-TARGETplus siRNA against SORLA #4 (CCACGUGUCUGCCCAAUUA)	Dharmacon	J-004722-05
siRNA against SORLA 3'UTR (sense strand: GGUUGGAGUGCCAAUAGAATT)	Qiagen	SI05039888
ON-TARGETplus siRNA against HER2 #2 (UGGAAGAGAUCACAGGUUA)	Dharmacon	J-003126-17
ON-TARGETplus siRNA against HER2 #4 (GCUCAUCGCUCACAACCAA)	Dharmacon	J-003126-20
Allstars Neg. ctrl (siCTRL)	Qiagen	1027281
pGFP-C-shLenti shSORL1 #1 (GGACTGGTCTGATGAGAAGGATTGTGGAG)	Origene	TL309181A
shSORL1 – GFP #4 (GTACATCTCTAGCAGTGCTGGAGCCAGGT)	Origene	TL309181C
pGFP-C-shLenti Scramble (shCTRL)	Origene	TR30021

Supplementary Table 2. Oligonucleotide sequences of siRNA and shRNA used in this study.
