# Supplementary material for: SORLA regulates endosomal trafficking and oncogenic fitness of HER2
Source: Nat Commun. 2019 May 28;10:2340. doi: 10.1038/s41467-019-10275-0 (PMC6538630; doi:10.1038/s41467-019-10275-0)
Supplement: Supplementary file 3 — Description of Additional Supplementary Files [file 41467_2019_10275_MOESM3_ESM.pdf]

Description of Additional Supplementary Files

File Name: Supplementary Movie 1
Description: Live-cell TIRF plane imaging of SORLA-GFP and AlexaFluor-568-labeled trastuzumab (Tz-568; red) in MDA-MB-361 cells.

File Name: Supplementary Movie 2
Description: Live-cell confocal imaging of SORLA-GFP and AlexaFluor-568-labeled trastuzumab (Tz-568; red) in the cytoplasm of MDA-MB-361 cells.
